# Data fairness—the new social challenge of the data economy: what shapes social media users’ perception of data fairness?

**DOI:** 10.3389/fpsyg.2026.1739496

**Published:** 2026-07-30

**Authors:** Chaoguang Huo, Fanfan Huo, Dongxiang Zhao

**Affiliations:** 1 School of Information Resource Management, Renmin University of China, Beijing, China; 2 Institute of Scientific and Technical Information of China, Haidian, China; 3 School of Information Management, Zhengzhou University, Zhengzhou, China

**Keywords:** algorithm awareness, data fairness, data privacy, data transparency, perceived data fairness, social media

## Abstract

**Background:**

Data fairness has become an increasingly important issue in the digital economy. Although social media platforms extensively collect and utilize personal data, limited research has examined how users perceive data fairness and the factors shaping such perceptions.

**Methods:**

Drawing on the Theory of Planned Behavior and Privacy Calculus Theory, a research model was developed incorporating algorithmic awareness, algorithmic attitude, data transparency, perceived control, privacy self-efficacy, and privacy concerns. Survey data from 589 WeChat users were analyzed using partial least squares structural equation modeling.

**Results:**

The results showed that algorithmic awareness positively influenced algorithmic attitude, which subsequently enhanced perceived data fairness. Data collection transparency and data processing transparency positively affected perceived control, whereas data use transparency had no significant effect. Perceived control and privacy self-efficacy positively influenced perceived data fairness. Privacy self-efficacy also reduced privacy concerns, which negatively affected perceived data fairness.

**Conclusion:**

This study advances the emerging literature on data fairness by identifying the key factors shaping users’ perceptions of data fairness in social media. The findings provide new insights into the psychological mechanisms underlying fairness perceptions and offer practical implications for developing more transparent and user-centered data governance practices.

## 1 Introduction

With the rapid development of the data-driven economy, algorithmic decision-making, and artificial intelligence, data fairness has arisen as one of the most significant social challenges of the digital age. Similar to education fairness and healthcare fairness, data fairness has become a fundamental prerequisite for promoting social justice and sustainable digital development (Micklitz et al., 2024; Zhang et al., 2023; Seastedt et al., 2022). As data increasingly influence how information is distributed, opportunities are allocated, and decisions are made, concerns regarding the fairness of data practices have expanded beyond technical communities to become a broader societal issue.

The growing importance of data fairness stems from the pervasive role of data throughout contemporary society (Meng, 2026; Huo and Zhao, 2026; Abiteboul and Stoyanovich, 2019; Taylor, 2019). Data are no longer merely resources for technological innovation; they shape social interactions, economic opportunities, and public decision-making processes. However, data collection and processing practices may inadvertently reproduce or amplify existing social inequalities. When certain groups are systematically underrepresented or misrepresented in datasets, algorithmic systems trained on such data may generate discriminatory outcomes and reinforce existing disparities (Goel et al., 2021). For example, through an analysis of 1.4 million images and videos collected from platforms like Google, Wikipedia, and YouTube, together with several large language models, Guilbeault et al. (2025) found that systematic distortions related to gender and age are deeply embedded within digital data ecosystems and may subsequently be amplified through algorithmic processing. These findings suggest that fairness concerns may emerge throughout the entire data lifecycle, from data collection and processing to algorithmic decision-making, highlighting the growing importance of data fairness in the digital age.

Consequently, data fairness should not be viewed solely as a technical or regulatory issue. Rather, it represents a multidimensional social challenge involving ethical principles, governance mechanisms, individual rights, and public trust. Importantly, fairness is inherently subjective. Individuals may interpret and evaluate the fairness of identical data practices differently depending on their experiences, values, expectations, and social contexts. Therefore, understanding how users perceive data fairness constitutes an essential prerequisite for promoting fair and responsible data governance.

The issue is particularly salient in social media environments. Social media platforms have become deeply embedded in everyday life, reshaping communication, information acquisition, social interaction, and public discourse (Huo and Huo, 2024). At the same time, these platforms continuously collect, process, and utilize vast quantities of user-generated data to support personalized services, targeted advertising, recommendation systems, and content moderation. While such practices create substantial value for both users and platform providers, they also raise growing concerns regarding privacy, transparency, accountability, and fairness (Daviet et al., 2022; Jain et al., 2021; Wu, 2024). Social media algorithms are frequently trained on large-scale user datasets that may reflect existing societal biases, potentially leading to unfair outcomes in recommendation, content visibility, advertising allocation, and information exposure (Scalvini, 2023; Shaham et al., 2022; Wang et al., 2025). As a result, perceptions of data fairness increasingly influence users’ trust in platforms, platform legitimacy, and broader public attitudes toward data-driven technologies.

Despite the growing attention devoted to fairness in digital environments, important gaps remain in current research. Existing studies have primarily examined fairness from technical, regulatory, or organizational perspectives (Colquitt, 2001), focusing on issues such as algorithmic fairness, bias mitigation, transparency, and governance mechanisms. Comparatively little attention has been paid to how ordinary users perceive the fairness of data practices themselves. Moreover, prior research frequently treats fairness as a broad concept encompassing algorithmic decision-making, content moderation, and platform governance, without clearly distinguishing perceived data fairness from related fairness constructs. Consequently, the psychological mechanisms through which users form judgments regarding the fairness of data collection, processing, and utilization practices remain insufficiently understood.

To address these gaps, this study conceptualizes perceived data fairness as a distinct psychological construct and investigates the factors shaping social media users’ perceptions of data fairness. Drawing upon the Theory of Planned Behavior and Privacy Calculus Theory, an integrated theoretical framework is developed to explain how transparency, perceived control, privacy-related perceptions, and algorithm-related perceptions jointly influence perceived data fairness. Specifically, this study seeks to answer the following research questions:

*RQ1*: What factors influence users’ perceptions of data fairness on social media?

*RQ2*: How do these factors shape user’s perceptions of data fairness on social media?

By answering these questions, this study contributes to the emerging literature on data fairness by extending existing research from technical and governance perspectives to the user perspective. It also provides practical insights for social media platforms seeking to enhance fairness, transparency, and user trust in data-driven environments.

## 2 Literature review

### 2.1 Data fairness

Data fairness has become an increasingly important issue in the digital economy as data-driven technologies are widely applied in social media, healthcare, finance, education, and public governance (Huo and Zhao, 2026). Although no universally accepted definition exists, data fairness generally refers to the equitable, transparent, accountable, and non-discriminatory treatment of individuals throughout the data lifecycle, including data collection, storage, processing, sharing, and utilization (Abiteboul and Stoyanovich, 2019; Jagadish et al., 2022; Meng, 2026).

Early studies primarily approached data fairness from a technical perspective, focusing on identifying and mitigating bias in datasets, algorithms, and machine learning systems (Anzum et al., 2022; Chen et al., 2023). Recent research has expanded beyond technical considerations and increasingly views data fairness as a governance issue involving transparency, accountability, stakeholder participation, and responsible data stewardship (Huo and Huo, 2023; Novelli et al., 2023; Singhal et al., 2024). Within this perspective, data fairness is not only reflected in algorithmic outcomes but also embedded in the procedures through which data are collected, processed, and used.

Despite these advances, most existing studies conceptualize data fairness as an objective characteristic that can be evaluated through technical indicators or governance standards Slamkov et al. (2022). However, data fairness is also inherently subjective. Because users often lack access to the technical details of data systems, their judgments are largely formed through personal experiences, expectations, and contextual interpretations. Consequently, objectively fair systems may not necessarily be perceived as fair by users (Starke et al., 2022). This observation highlights the importance of examining data fairness from the user perspective.

### 2.2 Perceived data fairness in social media

Social media platforms provide a particularly important context for investigating perceived data fairness because users continuously generate personal data through content creation, social interaction, and information sharing. These data are subsequently collected, processed, analyzed, and utilized by platform operators, making users frequent evaluators of platform data practices (Ohme et al., 2024; Wang et al., 2020; Kennedy et al., 2017). As data-driven technologies increasingly shape content distribution, advertising delivery, and platform governance, social media users’ evaluations of whether these data practices are fair have become a critical issue for both platform legitimacy and digital social equity.

Building upon prior data fairness research, perceived data fairness is defined in this study as users’ evaluations of whether data collection, processing, utilization, and governance practices are conducted in a fair, transparent, and equitable manner. Unlike objective assessments based on technical standards or regulatory requirements, perceived data fairness reflects users’ subjective judgments regarding the legitimacy and appropriateness of platform data practices. It is also conceptually distinct from algorithmic fairness. While algorithmic fairness focuses on whether algorithmic decisions and outcomes are unbiased and equitable, perceived data fairness concerns the fairness of the underlying data practices that support algorithmic systems. Because algorithmic systems fundamentally rely on data resources, data fairness can be regarded as an important foundation of algorithmic fairness (Chen et al., 2023; Meng, 2026).

Existing studies suggest that users’ data fairness evaluations are shaped by both individual and platform-related factors. At the individual level, privacy concerns, self-efficacy, trust, and digital literacy influence how users interpret and evaluate platform data practices (Dinev and Hart, 2006; Masur, 2020; Leonelli et al., 2021). At the platform level, transparency, accountability, explainability, perceived control, and algorithm-related perceptions have been identified as important antecedents of fairness evaluations (Martin, 2016; Shin, 2021; Starke et al., 2022). Recent studies further indicate that perceived data fairness plays a crucial role in shaping attitudes and acceptance toward AI-driven and data-driven platforms (Li and Zheng, 2024; Zhu and Liu, 2024; Jaoua, 2025).

Despite growing scholarly interest, perceived data fairness remains conceptually underdeveloped and empirically under-explored. First, existing studies frequently subsume data-related fairness concerns under broader concepts such as algorithmic fairness or platform fairness, resulting in limited conceptual clarity. Second, empirical research focusing specifically on perceived data fairness in social media environments remains scarce. Third, prior studies have largely examined transparency, privacy perceptions, perceived control, and algorithm-related perceptions separately, with limited efforts to integrate these factors within a unified explanatory framework. To address these gaps, this study conceptualizes perceived data fairness as an independent user-centered construct and develops an integrated model based on the Theory of Planned Behavior and Privacy Calculus Theory to explain how social media users evaluate the data fairness of social media platform’s data practices.

## 3 Theoretical basis and hypothesis development

### 3.1 Theoretical integration of TPB and Privacy Calculus Theory

The Theory of Planned Behavior (TPB) and Privacy Calculus Theory (PCT) provide complementary perspectives for understanding how users develop perceptions of data fairness in social media environments. Although both theories have traditionally been applied to explain behavioral intentions and information disclosure decisions, their underlying mechanisms are also highly relevant to individuals’ evaluations and judgments regarding digital platform practices.

TPB proposes that individuals form judgments and behavioral intentions based on cognitive evaluations of relevant objects and situations (Ajzen, 1991). While TPB is commonly used to explain behavioral intentions, its core constructs—attitude and perceived behavioral control—have also been widely employed to understand how individuals evaluate technologies, systems, and organizational practices (Maheshwari and Kha, 2022; Youngcharoen et al., 2017). In the context of social media, users continuously evaluate algorithmic operations and data governance practices. Their attitudes toward platform algorithms and their perceived ability to control personal data therefore constitute important cognitive foundations for fairness judgments. From this perspective, TPB provides a cognitive-evaluation framework explaining how users interpret and assess the fairness of platform data practices.

Privacy Calculus Theory offers a complementary perspective. PCT suggests that individuals evaluate digital services by balancing perceived benefits against privacy-related risks (Culnan and Armstrong, 1999; Dinev and Hart, 2006; Xu et al., 2009). Although originally developed to explain information disclosure behavior, subsequent studies have demonstrated that privacy-related evaluations also influence users’ trust, acceptance, and perceptions of digital platforms (Masur, 2020). In social media environments, users not only assess the benefits generated through data utilization but also evaluate potential privacy risks and their ability to manage those risks. Privacy self-efficacy and privacy concerns therefore represent two important mechanisms through which users evaluate the fairness of data practices.

Perceived data fairness is fundamentally a subjective evaluation of whether data collection, processing, utilization, and governance practices are conducted in a fair, transparent, and equitable manner. Such evaluations are shaped not only by users’ assessments of platform transparency, algorithms, and controllability, but also by their perceptions of privacy-related risks and their confidence in managing personal information. Consequently, neither TPB nor PCT alone can fully explain the formation of perceived data fairness.

In this study, TPB is employed to explain the cognitive evaluation process underlying fairness judgments, whereas PCT is used to explain the privacy-related evaluation process. The integration of these two theories enables a more comprehensive understanding of how social media users develop perceptions of data fairness. Based on this integrated framework, algorithmic awareness, algorithmic attitude, transparency, and perceived control are derived from the TPB perspective, while privacy self-efficacy and privacy concerns are derived from the PCT perspective.

### 3.2 Algorithmic awareness, algorithmic attitude, and perceived data fairness

Algorithmic awareness (AAW) refers to users’ awareness and understanding of how social media platforms employ algorithms to collect, process, analyze, and utilize personal data for functions such as content recommendation, information filtering, and targeted advertising (Dogruel et al., 2022; Zarouali et al., 2021). As algorithms increasingly mediate users’ access to information and shape online experiences, algorithmic awareness has become an important factor influencing how users interpret and evaluate platform operations. Users with higher levels of algorithmic awareness are generally more capable of recognizing the mechanisms through which algorithms influence content exposure, user interactions, and data-driven decision-making.

Algorithmic attitude (AAT) refers to individuals’ overall evaluative orientation toward the use of algorithms in social media enviroments (Gran et al., 2021; Lee, 2018). It reflects the extent to which users perceive algorithmic systems as useful, trustworthy, acceptable, and beneficial. Prior research suggests that users’ attitudes toward algorithms play an important role in shaping their evaluations of algorithm-mediated services and platform governance practices (Jago and Laurin, 2022; Logg et al., 2019).

According to the Theory of Planned Behavior (TPB), attitudes are formed through individuals’ cognitive evaluations of relevant objects and technologies (Ajzen, 1991). Greater knowledge and understanding generally reduce uncertainty and enable individuals to make more informed judgments regarding technological systems. Previous research suggests that users’ perceptions of algorithmic systems are closely associated with their understanding of how algorithms operate and influence online experiences (Bucher, 2017). In the context of social media, users who are more aware of how algorithms function may better understand the benefits and purposes of algorithmic decision-making, including personalized content delivery, information management, and service optimization. Such understanding can foster more favorable evaluations of algorithmic systems. Empirical studies have similarly shown that higher levels of algorithmic literacy and awareness contribute to more positive evaluations and greater acceptance of algorithmic technologies (Dogruel et al., 2022). Therefore, users with higher levels of algorithmic awareness are expected to hold more positive attitudes toward platform algorithms.

*H1*: Algorithmic awareness positively affects algorithmic attitude.

Perceived data fairness (PDF) refers to users’ subjective evaluation of whether personal data are collected, processed, and utilized in a fair, transparent, and equitable manner. Unlike objective assessments of fairness, it reflects users’ judgments regarding the legitimacy and appropriateness of platform data practices based on their experiences and expectations.

According to TPB, attitudes serve as an important cognitive basis for individuals’ evaluations and judgments (Ajzen, 1991). In social media environments, algorithms play a central role in data collection, processing, and utilization. Users who hold positive attitudes toward platform algorithms are more likely to perceive algorithmic operations as trustworthy, transparent, and beneficial, which in turn promotes favorable evaluations of the underlying data practices. Prior research has similarly shown that trust in algorithmic systems is associated with perceptions of transparency and procedural fairness (Starke et al., 2022; Shin, 2021). Therefore, a positive algorithmic attitude is expected to enhance perceived data fairness.

*H2*: Algorithmic Attitude positively affects perceived data fairness.

### 3.3 Data transparency, perceived control, and perceived data fairness

Perceived control (PCL) refers to individuals’ perception of their ability to access, manage, and regulate the collection and use of their personal data (Martin et al., 2017; Mothersbaugh et al., 2012). In social media environments, perceived control reflects the extent to which users believe they can determine what information is collected, how it is processed, and how it is subsequently used by platforms.

Data transparency has been widely recognized as a fundamental principle of responsible data governance and an important mechanism for reducing information asymmetry between users and digital platforms (Wong et al., 2023; Meng, 2026). According to Privacy Calculus Theory, individuals evaluate digital services by weighing potential benefits against privacy-related risks under conditions of incomplete information (Culnan and Armstrong, 1999; Dinev and Hart, 2006). When users lack information regarding platform data practices, uncertainty increases and their ability to make informed privacy-related decisions becomes constrained. Conversely, data transparency provides users with greater visibility into data-related activities, enabling them to better understand and evaluate platform data practices.

The relationship between data transparency and perceived control can also be explained through the Theory of Planned Behavior. Ajzen (1991) argued that perceived behavioral control is shaped by the availability of resources and information necessary for decision-making. In digital environments, data transparency functions as a critical informational resource that enables users to understand how their personal data are handled and to assess available privacy management options. Recent studies similarly suggest that data transparency enhances users’ sense of agency, empowerment, and control by making data practices more understandable and predictable (Martin et al., 2017; Wong et al., 2023; Ohme et al., 2024).

Data collection transparency (DCT) refers to the extent to which platforms clearly disclose what personal information is collected and under what conditions (Awad and Krishnan, 2006). Data processing transparency (DPT) concerns the openness of platform practices regarding how collected data are analyzed, stored, and shared (Martin et al., 2017). Data use transparency (DUT) refers to the extent to which users understand how their personal data are ultimately utilized by the platform (Martin et al., 2017; Awad and Krishnan, 2006). Because data transparency throughout the data lifecycle reduces uncertainty and improves users’ understanding of platform practices, it is expected to strengthen perceived control over their personal data. Therefore, the following hypotheses are proposed:

H3(a): Data collection transparency positively affects perceived control.

*H3(b)*: Data processing transparency positively affects perceived control.

*H3(c)*: Data use transparency positively affects perceived control.

Perceived control is further expected to influence perceived data fairness. In digital environments, perceived control represents an important form of user participation and agency because it reflects individuals’ ability to influence how their personal information is managed. When users believe they can access, modify, restrict, or otherwise influence the use of their personal data, they are more likely to regard platform data practices as legitimate, respectful, and equitable. Recent studies on AI fairness, platform governance, and social media fairness have similarly highlighted the importance of user agency and control in shaping data-related fairness evaluations (Zhu and Liu, 2024; Li and Zheng, 2024; Jaoua, 2025). Users who perceive greater control tend to evaluate data practices more positively because perceived control reduces feelings of vulnerability and power asymmetry while enhancing perceptions of data transparency, accountability, and procedural justice. Conversely, when users feel powerless in relation to platform data practices, they are more likely to perceive such data practices as opaque, exploitative, or unfair. Therefore, perceived control is expected to play a critical role in the formation of perceived data fairness. Therefore, the following hypothesis is proposed:

*H4*: Perceived control positively affects perceived data fairness.

### 3.4 Privacy self-efficacy, privacy concerns, and perceived data fairness

Privacy self-efficacy (PSE) refers to an individual’s confidence in his or her ability to manage privacy settings, control personal information and protect privacy in digital environments (Crossler and Bélanger, 2019; Larose and Rifon, 2007). Individuals with high level of privacy self-efficacy generally possess stronger confidence in their capability to cope with privacy-related risks and to make informed decisions regarding personal information disclosure.

From the perspective of Privacy Calculus Theory, users evaluate platform data practices based on their perceived ability to manage privacy risks. Individuals with higher privacy self-efficacy are more likely to understand privacy mechanisms, actively engage in privacy management, and feel empowered when interacting with social media platforms. As a result, they may be more likely to perceive platform data practices as fair and manageable. Recent studies have similarly shown that privacy self-efficacy positively influences individuals’ trust and evaluations of digital services (Crossler and Bélanger, 2019; Liu et al., 2024). Therefore, the following hypothesis is proposed:

*H5*: Privacy self-efficacy positively affects perceived data fairness.

Privacy concerns (PCC) refer to individuals’ worries about the collection, storage, use, and potential misuse of their personal information by digital platforms (Gerber et al., 2018; Masur, 2020). Within the privacy calculus perspective, privacy concerns represent users’ perceptions of privacy-related risks associated with platform data practices. Recent studies suggest that users increasingly evaluate digital platforms not only based on the benefits they provide but also on whether personal data are handled in a transparent, responsible, and trustworthy manner (Wong et al., 2023; Novelli et al., 2023). When users perceive a higher likelihood of unauthorized data sharing, misuse, or privacy violations, they are more likely to question the legitimacy of platform data practices and to evaluate such practices as less fair (Singhal et al., 2024; Huo and Zhao, 2026). Consequently, heightened privacy concerns may undermine users’ perceptions that data collection, processing, and utilization are conducted in a fair and equitable manner.

Although both transparency and privacy concerns are related to users’ evaluations of platform data practices, they represent distinct theoretical mechanisms. Transparency reflects users’ perceptions of information disclosure and openness regarding platform data activities, whereas privacy concerns capture users’ perceptions of potential risks associated with personal data misuse, unauthorized access, or privacy violations. From the perspective of Privacy Calculus Theory, transparency primarily reduces information asymmetry and enhances users’ sense of control, whereas privacy concerns represent risk perceptions that directly influence users’ evaluations of data practices. Therefore, these constructs capture different aspects of users’ cognitive evaluation processes rather than overlapping dimensions of the same construct.

Privacy self-efficacy may also reduce privacy concerns. Individuals who possess greater confidence in their ability to manage privacy settings and protect personal information are less likely to perceive privacy-related threats as uncontrollable. Prior studies consistently report that privacy self-efficacy mitigates perceived privacy risks and privacy concerns in online environments by enhancing individuals’ confidence in managing personal information and privacy settings (Liu et al., 2022; Crossler and Bélanger, 2019). Therefore, the following hypotheses are proposed:

*H6*: Privacy self-efficacy negatively affects privacy concerns.

*H7*: Privacy concerns negatively affects perceived data fairness.

**Figure 1:**
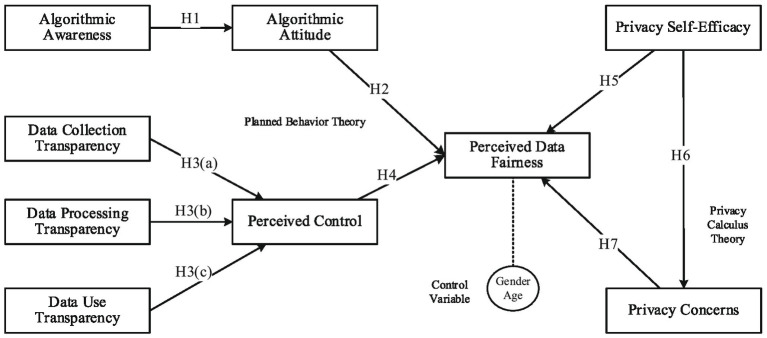
Research model of perceived data fairness in social media.

Based on the theoretical foundation and the proposed hypotheses, the research model shown in Figure 1 was developed to explain the formation mechanism of perceived data fairness in social media environments.

## 4 Research design

### 4.1 Variables and measurement

To ensure the validity of the questionnaire, all measurement scales were adapted from established instruments reported in previous studies and subsequently refined to fit the specific context of perceived data fairness in social media environments. The four measurement items of perceived data fairness (PDF) were adapted from Ambrose and Schminke (2009) and Cropanzano et al. (2007), with sample items including ‘I think it is fair for social media to collect my personal data’ and ‘I think it is fair for social media to process and analyze my data’. The five items on Algorithmic awareness (AAW) were adapted from Zarouali et al. (2021), with sample items including ‘Social media platforms employ algorithms for personalized content recommendations’. The three items on Algorithmic Attitude (AAT) were adapted from Gran et al. (2021) and Lee (2018), with sample items including ‘I trust the results produced by algorithmic operations and think the outcomes of algorithmic operations are value-neutral and objective’. The three items on data collection transparency (DCT), data processing transparency (DPT), data use transparency (DUT) were adapted from Martin et al. (2017) and Awad and Krishnan (2006) with sample items including ‘The social media’s customer data collection activities are: unclear to me or clear to me’ and ‘The social media’s customer data processing activities are: difficult to understand or easy to understand’. The four items on perceived control (PCL) were adapted from Martin et al. (2017) and Mothersbaugh et al. (2012), with sample items including ‘I believe I have control over what happens to my personal data’. The four items on privacy concerns (PCC) were adapted from Sutanto et al. (2013) and Dinev and Hart (2006), with sample items including ‘I am concerned that social media are collecting a lot of my personal data without my consent’. The three items on privacy self-efficacy (PSE) were adapted from Crossler and Bélanger (2019) and Larose and Rifon (2007), with sample items including ‘I can browse and understand the privacy policies provided by social media’. All variables in the questionnaire were measured using a 7-point Likert scale, ranging from 1 (strongly disagree) to 7 (strongly agree).

After the initial design of the questionnaire, revisions were made to certain measurement items based on feedback from domain experts and experienced social media users, resulting in a preliminary survey questionnaire. A pretest was subsequently conducted with 50 social media users recruited through online social networking platforms, including WeChat, TikTok and RedNote. Among the participants, 24 were male (48.0%) and 26 were female (52.0%). In terms of age, 31 participants (62.0%) were under 30 years old, while 19 participants (38.0%) were aged 31 years or above. The participants also represented diverse educational backgrounds and occupations. The pretest was administered online using the same survey platform employed in the formal study. Participants were asked not only to complete the questionnaire but also to provide feedback regarding item clarity, wording, readability, and questionnaire length. The pretest results showed that all constructs achieved Cronbach’s alpha values above 0.70, indicating satisfactory internal consistency. However, several respondents reported that some items were repetitive or difficult to interpret in the social media context. Based on participants’ feedback and the pretest results, the order and wording of several items were refined, and items with ambiguous or unclear meanings were removed. For example, in the Perceived Data Fairness scale, the original item “I think it is fair for social media to process my data” was revised to “I think it is fair for social media to process and analyze my data” to better capture contemporary data-driven practices. Similarly, the original item “I think it is fair for social media to use my data” was revised to “I think it is fair for social media to share and utilize my data” to more comprehensively reflect users’ concerns regarding data circulation and third-party access. The questionnaire (see Appendix A) was then finalized for the formal survey.

### 4.2 Questionnaire collection

The formal questionnaire was then distributed to a broader target population via WeChat, one of the most widely used social media platform, with approximately 1.3 billion monthly active users worldwide. Prior to participation, respondents were informed of the purpose of the study and their rights as participants. Participation was voluntary and anonymous, and electronic informed consent was obtained from all respondents before they proceeded to the questionnaire. Respondents could discontinue the survey at any time without penalty, and no personally identifiable information was collected during the survey.

Data collection was conducted over a 60-day period from December 2023 to January 2024. A total of 665 questionnaires were received. After excluding invalid responses and questionnaires completed in an unusually short amount of time, 589 valid questionnaires were retained for analysis, yielding a valid response rate of 88.6%. Among the valid respondents, 47.4% were male and 52.6% were female. The demographic characteristics of the sample are presented in Table 1.

**Table 1:** Demographic characteristics of the research sample.

Characteristics	Range	Frequency	Percentage (%)
Gender	Male	279	47.4
	Female	310	52.6
Age	20 or below	16	2.7
	21–30	268	45.5
	31–40	251	42.6
	41–50	44	7.5
	51 or above	10	1.7
Education Level	High school or below	17	2.9
	Junior college	28	4.7
	Undergraduate	471	80.0
	Master or above	73	12.4

## 5 Data analysis and results

### 5.1 Reliability and validity analysis

Reliability and validity analyses were conducted to assess the quality of the measurement model. Composite reliability (CR) and Cronbach’s alpha were used as indicators of internal consistency reliability. Values exceeding 0.70 for both CR and Cronbach’s alpha indicates satisfactory reliability (Gefen and Straub, 2005). Convergent validity was evaluated using item loading and average variance extracted (AVE) (Gefen and Straub, 2005).

As shown in Table 2, all item loadings exceed the recommended threshold of 0.70, demonstrating that the items adequately represented their corresponding latent variables. Cronbach’s alpha values ranged from 0.726 to 0.901, while CR values ranged from 0.846 to 0.931, both exceeding the recommended cutoff value of 0.70. In addition, all AVE values were above 0.50, demonstrating satisfactory convergent validity. Discriminant validity was assessed using the Fornell-Larcker criterion. As reported in Table 3, the square root of the AVE for each variable was greater than its correlations with all other variables, indicating adequate discriminant validity (Huo et al., 2018; Gefen and Straub, 2005).

**Table 2:** Reliability and convergent validity of the latent variables.

Latent variables	Items	Loadings	Outer VIF	Cronbach’s alphas	CR	AVE
Algorithmic awareness	AAW1	0.747	1.459	0.814	0.870	0.573
	AAW2	0.758	1.621			
	AAW3	0.764	1.554			
	AAW4	0.735	1.680			
	AAW5	0.779	1.544			
Algorithmic attitude	AAT1	0.820	1.448	0.803	0.864	0.560
	AAT2	0.804	1.477			
	AAT3	0.789	1.291			
Data collection transparency	DCT1	0.828	1.636	0.726	0.846	0.646
	DCT2	0.818	1.525			
	DCT3	0.764	1.270			
Data processing transparency	DPT1	0.871	1.853	0.850	0.909	0.769
	DPT2	0.881	2.205			
	DPT3	0.879	2.231			
Data use transparency	DUT1	0.845	1.658	0.807	0.886	0.722
	DUT2	0.872	1.660			
	DUT3	0.831	1.570			
Perceived control	PCL1	0.801	2.093	0.840	0.889	0.667
	PCL2	0.813	2.283			
	PCL3	0.847	2.541			
	PCL4	0.806	1.455			
Privacy self-efficacy	PSE1	0.878	1.918	0.775	0.869	0.690
	PSE2	0.874	1.919			
	PSE3	0.733	1.239			
Privacy concerns	PCC1	0.861	2.734	0.900	0.930	0.768
	PCC2	0.858	2.339			
	PCC3	0.891	2.451			
	PCC4	0.893	2.690			
Perceived data fairness	PDF1	0.892	2.941	0.901	0.931	0.771
	PDF2	0.871	2.634			
	PDF3	0.876	2.693			
	PDF4	0.873	2.571			

CR, Composite Reliability; AVE, Average Variance Extracted; VIF, Variance Inflation Factor.

**Table 3:** Discriminant validity assessment using the Fornell–Larcker criterion.

Variable	PCL	DUT	PDF	DPT	DCT	AAT	AAW	PSE	PCC
PCL	**0.817**								
DUT	0.437	**0.85**							
PDF	0.472	0.606	**0.878**						
DPT	0.501	0.797	0.656	**0.877**					
DCT	0.478	0.754	0.604	0.727	**0.804**				
AAT	0.488	0.538	0.631	0.558	0.533	**0.748**			
AAW	0.238	0.278	0.273	0.29	0.304	0.467	**0.757**		
PSE	0.462	0.596	0.678	0.635	0.576	0.603	0.247	**0.831**	
PCC	−0.02	−0.17	−0.197	−0.191	−0.117	−0.049	0.054	−0.175	**0.876**

Diagonal values (bold) represent the square root of the average variance extracted (AVE) for each construct, whereas off-diagonal values represent inter-construct correlations. Discriminant validity is established when the square root of AVE is greater than the correlations between the focal construct and all other constructs (Fornell and Larcker, 1981).

### 5.2 Collinearity test and common method bias

Variance inflation factor (VIF) values were used to assess potential multicollinearity among the constructs. As shown in Table 2, the outer VIF values ranged from 1.239 to 2.941, while the Inner VIF values as shown in Table 4 ranged from 1.000 to 4.148. All VIF values were well below the recommended threshold of 10 (Petter et al., 2007), indicating that multicollinearity was not a concern in this study.

**Table 4:** Inner variance inflation factor (VIF) values.

Variable	PCL	AAT	PCC	PDF
DCT	3.226			
DPT	3.103			
DUT	4.148			
AAW		1.000		
AAT				1.994
PSE			1.000	1.996
PCC				1.036
PCL				1.498

To reduce the risk of common method bias, both ex-ante procedural controls and post-hoc tests were implemented during questionnaire design and data collection, including the use of validated measurement scales, expert review, pretesting, anonymous participation, and clear questionnaire instructions. In addition, Harman’s single-factor test was employed as a post-hoc assessment. The results showed that the first unrotated factor accounted for 31.72% of the total variance, which is below the commonly accepted threshold of 40% (Podsakoff et al., 2012; Podsakoff et al., 2003). Taken together, these results reveal that common method bias is unlikely to pose a significant issue in this study.

### 5.3 Results of hypothesis testing

**Figure 2:**
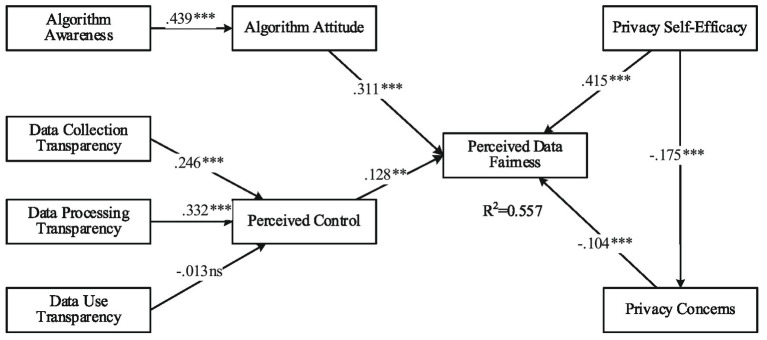
Results of the structural model.

Structural equation modeling (SEM) was performed using SmartPLS 3.0 software to test the proposed hypotheses. Evaluation metrics such as R^2^, SRMR, and NFI were utilized to assess the model’s explanatory power (Leguina, 2015). The results of the structural model and hypothesis tests are presented in Figure 2 and Table 5. As shown in Figure 2, the corresponding R^2^ values for perceived data fairness is 0.557, indicating moderate explanatory power. The standardized root mean square residual (SRMR) is 0.067, which is below the recommended threshold of 0.08, suggesting a satisfactory model fit (Wetzels et al., 2009). Overall, these results indicate that the proposed model provides an adequate representation of the observed data.

**Table 5:** Multiple regression results.

Hypothesis	Path coefficient	*t*-values	*p*-values	Standard error	Result
H1: AAW → AAT	0.439	8.992	0.000	0.049	Supported
H2: AAT → PDF	0.311	6.493	0.000	0.048	Supported
H3(a): DCT → PCL	0.246	3.358	0.000	0.073	Supported
H3(b): DPT → PCL	0.332	4.234	0.000	0.078	Supported
H3(c): DUT → PCL	−0.013	0.164	0.870	0.078	Not Supported
H4: PCL → PDF	0.128	3.024	0.003	0.042	Supported
H5: PSE → PDF	0.415	8.177	0.000	0.051	Supported
H6: PSE → PCC	−0.175	4.192	0.000	0.042	Supported
H7: PCC → PDF	−0.104	4.36	0.000	0.024	Supported
Model evaluation	R^2^	0.557 (≥0.33)			
	SRMR	0.067 (<0.08)			
	NFI	0.882 (≥0.80)			

Table 5 indicates that all hypotheses are supported except for H3(c). Algorithmic awareness (*β* = 0.439, t = 8.992, *p* < 0.001) positively and significantly affects Algorithmic Attitude, and Algorithmic Attitude (*β* = 0.311, t = 6.493, *p* < 0.001) has a significant impact on perceived data fairness, supporting H1 and H2. Data collection transparency (*β* = 0.246, t = 3.358, p < 0.001) and data processing transparency (*β* = 0.332, t = 4.234, *p* < 0.001) both have significant impact on perceived control, supporting H3(a) and H3(b), while data use transparency (*β* = −0.013, t = 0.164, *p* > 0.05) has an insignificant impact on perceived control, indicating H3(c) is rejected. Perceived control (*β* = 0.128, t = 3.024, *p* < 0.01) reveals a positive and significant impact on perceived data fairness, H4 is supported. Although privacy self-efficacy (*β* = 0.415, t = 8.177, p < 0.001) has a significant and positive impact on perceived data fairness (H5 is supported), it significantly and negatively affects privacy concerns (*β* = −0.175, t = 4.192, *p* < 0.001), and privacy concerns negatively affects perceived data fairness (*β* = −0.104, t = 4.36, *p* < 0.001), supporting H6 and H7. As for control variable, gender, age and education level have no significant influence on perceived data fairness.

## 6 Discussion

This study investigated how social media users form perceptions of data fairness by integrating the Theory of Planned Behavior (TPB) and Privacy Calculus Theory (PCT). The findings suggest that perceived data fairness is shaped by a combination of algorithm-related cognition, transparency perceptions, perceived control, and privacy-related beliefs. These results contribute to the growing literature on data fairness by highlighting the psychological mechanisms through which users evaluate the fairness of platform data practices.

A notable finding concerns the role of algorithm-related cognition. The results indicate that algorithmic awareness enhances algorithmic attitude, which subsequently contributes to higher levels of perceived data fairness. As social media platforms increasingly rely on algorithmic systems to curate content, personalize services, and allocate information resources, users rarely observe data practices directly. Instead, data fairness evaluations are often formed through users’ understanding and interpretation of algorithmic processes. Recent studies have shown that perceptions of algorithmic fairness play a crucial role in shaping users’ attitudes toward AI-enabled systems and digital platforms (Li and Zheng, 2024; Zhu and Liu, 2024). The present findings extend this line of research by suggesting that algorithm-related cognition influences not only evaluations of algorithms themselves but also broader judgments regarding the fairness of underlying data practices. This observation aligns with recent arguments that data fairness concerns originate from the entire data lifecycle rather than solely from algorithmic outputs (Chen et al., 2023; Meng, 2026). Consequently, social media users who better understand algorithmic systems may be more likely to perceive data collection, processing, and utilization practices as unbiased and equitable.

The findings also underscore the importance of data transparency and perceived control in shaping data fairness perceptions. Data collection transparency and data processing transparency significantly increased perceived control, whereas data use transparency did not exhibit a significant effect. Recent research on data governance has increasingly emphasized transparency as a prerequisite for fairness, accountability, and responsible data stewardship (Novelli et al., 2023; Singhal et al., 2024; Wong et al., 2023). The present findings partially support this view by demonstrating that data transparency enhances users’ sense of control when it provides meaningful information about how data are collected and processed. However, transparency regarding data use appears insufficient to generate the same effect. One possible explanation is that contemporary social media ecosystems involve highly complex downstream data practices, including algorithmic profiling, targeted advertising, and third-party data sharing. Although such practices are often disclosed, users may find the information difficult to understand or translate into actionable decisions. This interpretation is consistent with recent concerns that increasing complexity in platform data infrastructures has widened the gap between formal transparency and users’ actual understanding of data practices (Ohme et al., 2024). The findings therefore suggest that meaningful transparency may be more important than transparency alone.

Perceived control emerged as another important determinant of perceived data fairness. Existing studies have frequently examined perceived control as an antecedent of privacy disclosure, trust, or technology acceptance. The present study extends this literature by demonstrating its relevance to data fairness evaluations. Recent discussions on responsible AI and data governance increasingly argue that data-related fairness involves not only equitable outcomes but also opportunities for participation, agency, and influence over data-related processes (Wang et al., 2023; Agu et al., 2024; Novelli et al., 2023). From this perspective, users may perceive data practices as fair when they believe they retain meaningful control over how personal information is managed. The findings therefore suggest that data fairness perceptions are shaped not only by platform behavior but also by users’ subjective sense of empowerment.

Another important contribution concerns the relationship between privacy-related perceptions and perceived data fairness. Privacy self-efficacy was found to enhance perceived data fairness while simultaneously reducing privacy concerns. In contrast, privacy concerns negatively influenced data fairness perceptions. These findings indicate that data-related fairness judgments are closely intertwined with individuals’ assessments of privacy risks and coping capabilities. While prior studies grounded in Privacy Calculus Theory have primarily focused on information disclosure decisions, the present findings suggest that privacy-related evaluations also play a central role in data-related fairness formation. Recent scholarship increasingly emphasizes the interdependence of data fairness, privacy, transparency, and accountability within digital environments (Singhal et al., 2024; Meng, 2026; Chen et al., 2023). Consistent with this perspective, social media users who feel capable of managing privacy risks may be more inclined to view platform data practices as fair, whereas concerns regarding surveillance, misuse, or unauthorized access may undermine data fairness perceptions by signaling potential violations of users’ expectations regarding equitable treatment.

Overall, the findings contribute to the emerging literature on data fairness by demonstrating that perceived data fairness is jointly shaped by algorithm-related cognition, transparency-based governance mechanisms, perceived control, and privacy-related psychological factors. While recent studies have predominantly examined data fairness from technical, regulatory, or algorithmic perspectives (Chen et al., 2023; Meng, 2026; Kämpf et al., 2026), the present study highlights the importance of users’ subjective evaluations in shaping data fairness judgments. By integrating TPB and Privacy Calculus Theory, this study provides a user-centered explanation of how perceived data fairness is formed in social media environments and offers empirical evidence that data fairness perceptions extend beyond algorithmic outputs to encompass the broader data lifecycle.

## 7 Theoretical and management implications

### 7.1 Theoretical implications

This study contributes to the literature in three important ways. First, it advances data fairness research by shifting the focus from technical and institutional perspectives toward users’ subjective perceptions. Existing studies primarily conceptualize data fairness as an objective property of data systems, emphasizing algorithmic bias mitigation, fairness metrics, and governance mechanisms (Catania et al., 2023; Abiteboul and Stoyanovich, 2019; Gallese et al., 2025). The present study demonstrates that fairness also exists as a psychological perception shaped by users’ experiences, beliefs, and evaluations of platform practices. Second, this study enriches research on fairness perceptions in digital environments by conceptualizing perceived data fairness as a distinct construct. Previous studies have largely focused on algorithmic fairness, platform fairness, or procedural fairness (Starke et al., 2022; Shin, 2021). By distinguishing perceived data fairness from algorithmic fairness, the study provides a more nuanced understanding of how users evaluate fairness throughout the data lifecycle, including data collection, processing, and utilization. Third, this study contributes to theory development by integrating TPB and PCT to explain the formation of perceived data fairness. TPB explains how cognitive beliefs regarding algorithms and control mechanisms influence fairness evaluations, whereas PCT explains how privacy-related benefits and risks shape such evaluations. The integration of these perspectives provides a more comprehensive explanation of the cognitive and privacy-related mechanisms underlying perceived data fairness.

### 7.2 Management implications

The findings also offer several practical implications for social media platforms and policymakers. First, platforms should improve algorithmic transparency and strengthen users’ understanding of algorithmic operations. Providing accessible explanations regarding recommendation mechanisms and data processing practices may enhance algorithmic attitudes and ultimately improve perceptions of data fairness. Second, social media companies should prioritize transparency in data collection and processing procedures. Transparency measures should go beyond compliance-oriented disclosures and focus on providing understandable, actionable information that enhances users’ sense of control. Third, platforms should empower users through stronger privacy management tools and privacy education initiatives. Enhancing privacy self-efficacy can reduce privacy concerns while simultaneously improving perceptions of fairness. Finally, policymakers should encourage the development of user-centered data governance frameworks that emphasize transparency, accountability, and user empowerment. Such efforts may contribute to improving public trust in data-driven platforms and promoting a more equitable digital environment.

## 8 Limitations and future research

Although this study has produced several strong findings, it is subject to certain limitations that need to be addressed in future research. First, perceived data fairness remains a relatively new construct. Although measurement items were adapted from established scales and validated through empirical testing, future studies should continue refining and validating dedicated measurement instruments for perceived data fairness. Second, this study employed a cross-sectional survey design, which limits causal inference. Future research may adopt longitudinal or experimental approaches to examine how fairness perceptions evolve over time and under different platform conditions. Third, the sample was collected from Chinese social media users, primarily within the WeChat ecosystem. Cultural values, regulatory environments, and platform characteristics may influence fairness perceptions. Future studies should conduct cross-cultural comparisons to examine the generalizability of the proposed model. Fourth, the present study focused on algorithm-related, transparency-related, and privacy-related factors. As data governance practices continue to evolve, additional factors such as AI literacy, institutional trust, data ownership perceptions, and regulatory awareness may also influence perceived data fairness and deserve further investigation.

## 9 Conclusion

As data-driven technologies increasingly shape social interactions and decision-making processes, understanding how individuals evaluate the fairness of data practices has become an important research and governance challenge. This study developed and empirically tested an integrated model of perceived data fairness in social media by combining the Theory of Planned Behavior and Privacy Calculus Theory. The results demonstrate that algorithmic awareness, algorithmic attitude, perceived control, privacy self-efficacy, and privacy concerns play significant roles in shaping users’ perceptions of data fairness. The findings further reveal that transparency contributes to fairness perceptions primarily through enhancing users’ sense of control over personal data. By conceptualizing perceived data fairness as a distinct psychological construct and identifying its key antecedents, this study extends existing research on data fairness and provides practical insights for the design of more transparent, trustworthy, and user-centered data governance mechanisms in social media environments.

## Data Availability

The raw data supporting the conclusions of this article will be made available by the authors, without undue reservation.
