# Supplementary material for: Data fairness—the new social challenge of the data economy: what shapes social media users’ perception of data fairness?
Source: Front Psychol. 2026 Jul 30;17:1739496. doi: 10.3389/fpsyg.2026.1739496 (PMC13469658; doi:10.3389/fpsyg.2026.1739496)
Supplement: Supplementary file 1 [file Supplementary_file_1.docx]

**Appendix** A

***The*** ***measurement scales***

| Variables | Measurement Items | Sources |
| --- | --- | --- |
| Algorithmic Awareness (AAW) | AAW1: Social media platforms utilize algorithms to push content. | Zarouali et al. (2021) |
|  | AAW2: Social media platforms employ algorithms for personalized content recommendations. |  |
|  | AAW3: Algorithms on social media platforms automatically push content to users based on automated decisions. |  |
|  | AAW4: The algorithms on social media platforms recommend content to users based on their online behavioral data on the platform. |  |
|  | AAW5: The prioritization of content pushed on social media platforms involves algorithms. |  |
| Algorithmic Attitude (AAT) | AAT1: I think that algorithm-driven recommendations on social media platforms are effective in providing content of interest. They can guide me to discover new information and resources. | Gran et al (2021); Lee (2018) |
|  | AAT2: I think that algorithm-driven advertisements on social media platforms can provide me with products and services of interest. They can bring me new valuable information and experiences. |  |
|  | AAT3: I trust the results produced by algorithmic operations and think the outcomes of algorithmic operations are value-neutral and objective. |  |
| Data Collection Transparency (DCT) | The social media’s customer data collection activities are:   - DCT1: Unclear to me / Clear to me - DCT2: Difficult to understand / Easy to understand - DCT3: Vague / Transparent | Awad et al. (2006); Martin et al. (2017) |
| Data Processing Transparency (DPT) | The social media’s customer data processing activities are:   - DPT1: Unclear to me / Clear to me - DPT2: Difficult to understand / Easy to understand - DPT3: Vague / Transparent | Awad et al. (2006); Martin et al. (2017) |
| Data Use Transparency (DUT) | The social media’s customer data use activities are:   - DUT1: Unclear to me / Clear to me - DUT2: Difficult to understand / Easy to understand - DUT3: Vague / Transparent | Awad et al. (2006);  Martin et al. (2017) |
| Perceived Control (PCL) | PCL1: I believe I have control over what happens to my personal data. | Mothersbaugh et al. (2012); Martin et al. (2017) |
|  | PCL2: It is up to me how much the social media uses my data |  |
|  | PCL3: I have a say in how my data is used by the social media |  |
|  | PCL4: I have a say in whether my personal data is shared with others |  |
| Privacy Concerns (PCC) | PCC1: I am concerned that social media are collecting a lot of my personal data without my consent. | Sutanto et al. (2013); Dinev and Hart (2006) |
|  | PCC2: I am concerned that social media are using my personal information and data without my consent. |  |
|  | PCC3: I don't know how my personal data will be used, it scares me. |  |
|  | PCC4: I worry that platforms will track and monitor what I do in social media. |  |
| Privacy Self-efficacy (PSE) | PSE1: I can browse and understand the privacy policies provided by social media. | Larose and Rifon, (2007); Crossler and Bélanger (2019) |
|  | PSE2: I am able to effectively control and manage social media privacy Settings to protect my personal data. |  |
|  | PSE3: Overall, I am confident that I can protect my privacy in social media. |  |
| Perceived Data Fairness (PDF) | PDF1: I think it is fair for social media to collect my personal data. | Ambrose and Schminke (2009); Cropanzano et al. (2007) |
|  | PDF2: I think it is fair for social media to process and analyze my data. |  |
|  | PDF3: I think it is fair for social media to share and utilize my data. |  |
|  | PDF4: Overall, I am satisfied with the fairness of social media in collecting, processing, and utilizing my data. |  |
